# Treatment Algorithm for Ameloblastoma

**DOI:** 10.1155/2014/121032

**Published:** 2014-12-07

**Authors:** Madhumati Singh, Anjan Shah, Auric Bhattacharya, Ragesh Raman, Narahari Ranganatha, Piyush Prakash

**Affiliations:** Department of Oral and Maxillofacial Surgery, Raja Rajeswari Dental College, Bangalore 560074, India

## Abstract

Ameloblastoma is the second most common benign odontogenic tumour (Shafer et al. 2006) which constitutes 1–3% of all cysts and tumours of jaw, with locally aggressive behaviour, high recurrence rate, and a malignant potential (Chaine et al. 2009). Various treatment algorithms for ameloblastoma have been reported; however, a universally accepted approach remains unsettled and controversial (Chaine et al. 2009). The treatment algorithm to be chosen depends on size (Escande et al. 2009 and Sampson and Pogrel 1999), anatomical location (Feinberg and Steinberg 1996), histologic variant (Philipsen and Reichart 1998), and anatomical involvement (Jackson et al. 1996). In this paper various such treatment modalities which include enucleation and peripheral osteotomy, partial maxillectomy, segmental resection and reconstruction done with fibula graft, and radical resection and reconstruction done with rib graft and their recurrence rate are reviewed with study of five cases.

## 1. Introduction

Ameloblastoma is a true neoplasm of enamel organ type. Robinson described it as unicentric, nonfunctional, intermittent in growth, anatomically benign, and clinically persistent [1]. It is the second most common odontogenic neoplasm [1]. Histologically it is of six subtypes: follicular, plexiform, acanthomatous, granular, desmoplastic, and basilar [2]. It affects mandible more than maxilla especially in the region of molar-ramus area. It causes a slow growing, painless expansion of jaw which causes thinning of cortical plates. Root resorption, tooth mobility, and paresthesia are features seen in advanced cases of ameloblastoma. Radiographically it can be unicystic, multicystic, or solid and peripheral type [2]. Multicystic or solid type is prevalent in 86% of cases. Unicystic ameloblastoma is of three subtypes: luminal, intraluminal, and mural [3].

Treatment modalities are dictated by size [4, 5], anatomical location (Table 1) [6], histologic variant, and anatomical involvement [7]. On the one hand, there is a school advocating major segmental or en bloc resection for ameloblastoma with a requirement of 1–1.5 cm of clinically and radiographically normal bone and uninvolved margins. On the other hand, there is a school advocating a more conservative surgical management by enucleation with adjacent bone [4].

## 2. Case Reports

### 2.1. Case  1 (See Figure 1)

A 28-year-old male patient reported to the department with the chief complaint of pain in the left lower jaw region for the last three months. Extraoral examination revealed a diffuse hard swelling measuring approximately 3 cm × 2 cm. On intraoral palpation there was expansion of buccal and lingual cortical plates. Decompression and packing with BIPP paste were done to prevent pathological fracture. After 6 months enucleation with curettage was done. Incisional biopsy revealed unicystic mural ameloblastoma. The patient was operated on under LA. A regular follow-up is being done. There is no sign of recurrence.

### 2.2. Case  2 (See Figure 2)

A 17-year-old female patient reported to the department two years back with the chief complaint of swelling in the right lower jaw region for the last four months. On extraoral examination a nontender swelling approximately of the size 4 cm × 2.5 cm was appreciated in the left mandibular region extending from lateral incisor to lower third molar region. There was expansion of buccal and lingual cortical plates. Incisional biopsy revealed unicystic mural ameloblastoma.

The patient was operated on under GA. Lesion was completely enucleated. Impacted teeth (33, 34, 35, and 36) were extracted. Peripheral osteotomy was done. Primary closure was achieved. A regular follow-up is being done. There is no sign of recurrence.

### 2.3. Case  3 (See Figure 3)

A 25-year-old male patient reported to the department with the chief complaint of swelling in the lower left back tooth region for the last year. On extraoral examination we could palpate a swelling approximately of the size 6 cm × 3 cm extending from the commissure of lip to the posterior border of the mandible. On intraoral palpation there was expansion of buccal and lingual cortical plates and perforation of lingual cortical plates. Incisional biopsy was done. It revealed plexiform ameloblastoma. The patient was operated on under GA. Segmental resection with disarticulation of the left mandible was done followed by reconstruction with microvascular fibula free flap using reconstruction plate. A regular follow-up is being done. There is no sign of recurrence.

### 2.4. Case 4 (See Figure 4)

A 60-year-old male patient reported to the department of OMFS, Raja Rajeswari Dental college, Bangalore, with the chief complaint of swelling on left middle third of face for the past four months. On extraoral examination a diffuse swelling measuring approximately 5 × 4 cm was felt which extended from ala of nose to the tragus of ear and infraorbital margin to below the commissure of lip. On intraoral examination a bony hard swelling was present extending from midline to 1st premolar region and cervical margin to the nasal floor. Incisional biopsy was done. It revealed follicular type of ameloblastoma. Partial maxillectomy was done under general anaesthesia (Table 2). A regular follow-up is being done. There is no sign of recurrence.

### 2.5. Case 5 (See Figure 5)

A 28-year-old female patient reported to the department with the chief complaint of swelling in the lower left back tooth region for the last three months. On extraoral examination, there was a swelling approximately of the size 4 cm × 4 cm extending from left commissure of lip to the posterior border of ramus of mandible and from ala-tragus line to 1 cm below the lower border of mandible. On intraoral examination there was bony expansion in buccal and lingual cortical plate and perforation of lingual cortical plate. Incisional biopsy was done. It revealed follicular type of ameloblastoma. Segmental resection with disarticulation of the left mandible was done followed by reconstruction with rib graft using reconstruction plate. A regular follow-up is being done. There is no sign of recurrence.

## 3. Discussion

Treatment modalities are based on algorithms which are dictated by size [4, 5], anatomical location [6], histologic variant [3], and anatomical involvement [7].

**Figure 6:**
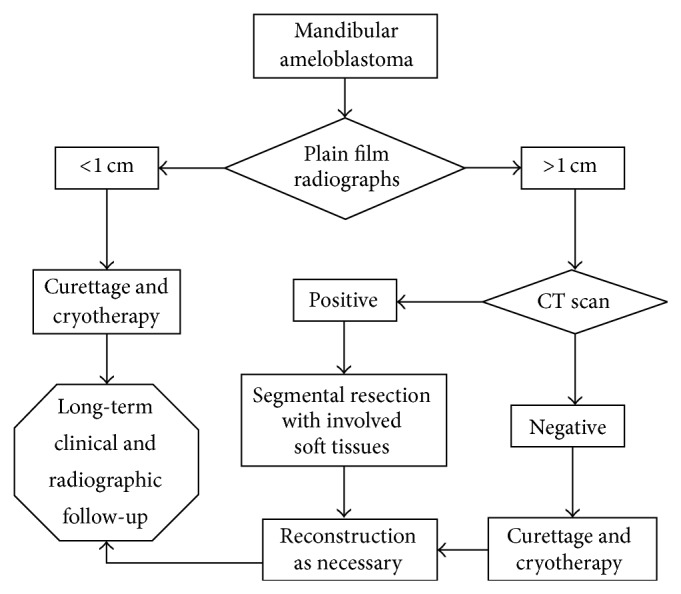


According to a retrospective study done in Northern California for both primary management and treatment of recurrences for mandibular ameloblastoma, specific diagnostic and treatment techniques had been applied which had resulted in satisfactory results. This has been refined into an algorithm (Figure 6) that allowed the clinician to have an organized approach to treating these tumours [5].

Based on a study done in Pitié-Salpêtrière Hospital from 1994 to 2007, 114 patients were studied and consequently divided into three groups: less than 5 cm, between 5 and 13 cm, and more than 13 cm (corresponding to the group of the giant ameloblastomas) [4]. Then, jaw locations were studied. Regarding site, the maxilla was divided into three regions: anterior, premolar, and molar areas. The mandible was divided into five areas: symphyseal, parasymphyseal, horizontal ramus, angle, vertical ramus, coronoid process, and cranial base [4].

**Figure 7:**
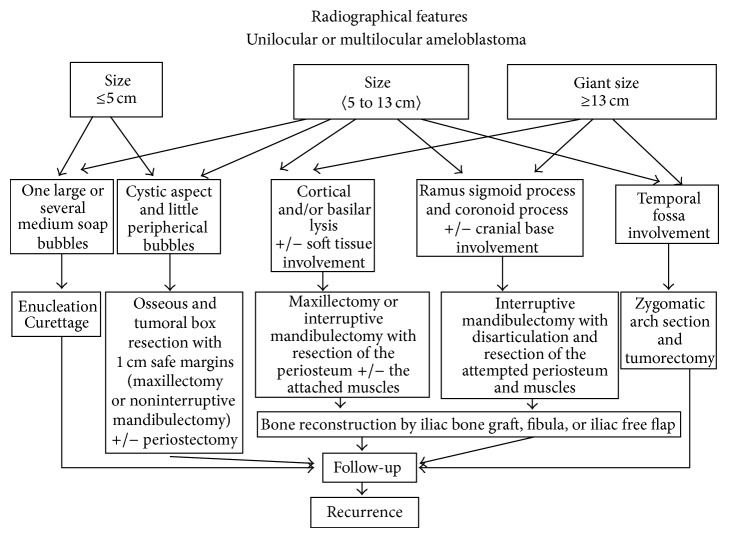


According to the results and considering the four main parameters (radiographic presentation, histologic type, size, and location), the study done in Pitie Salpeterie hospital proposed a therapeutic algorithm for ameloblastomas (Figure 7) [4].

Similarly based on a study done in the Institute of Craniofacial and Reconstructive Surgery, a treatment algorithm was developed for treating maxillary ameloblastoma based on anatomic involvement [7].

## 4. Conclusion

Treatment of a patient with an ameloblastoma should be based on accurate clinical details, radiographs, special imaging, and a representative biopsy, followed and reviewed by an oral pathologist and a maxillofacial surgeon. This study provides information about the therapeutic management of 5 adult cases of ameloblastoma, seen in our department. This study was based on a treatment algorithm for adult ameloblastomas based on radiographic appearance, histologic type, size, and location. Each case is unique and has to be considered in the clinical context and the relationship of the lesion to surrounding tissues, histological type, and recurrence rate. A minimum of ten years of follow-up is required in all the cases. It remains each clinician's responsibility to formulate an individual surgical plan for each patient: a therapeutic algorithm is just a guide.

## Figures and Tables

**Figure 1:**
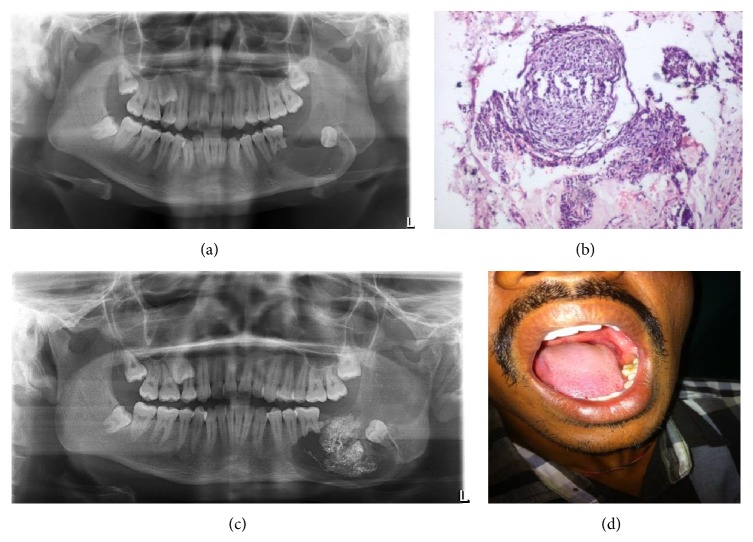
(a) Preoperative. (b) Histopathological slide. (c) Intraoperative. (d) Postoperative.

**Figure 2:**
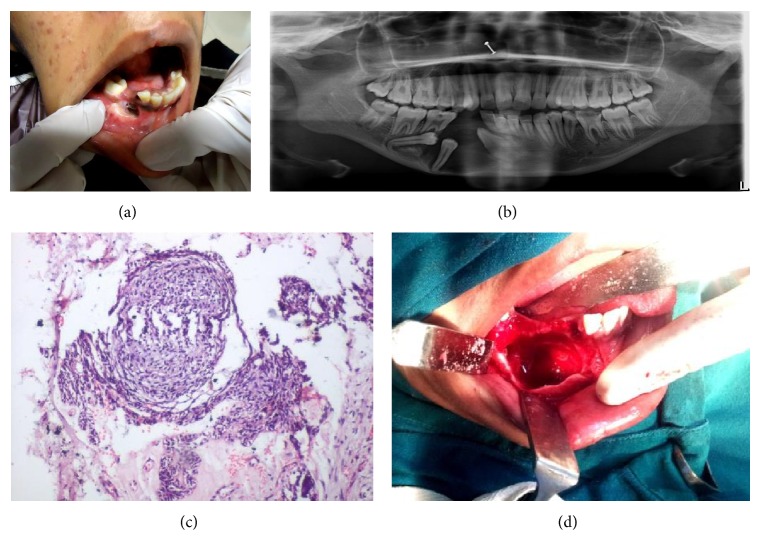
(a) Preoperative view. (b) OPG. (c) Unicystic mural ameloblastoma. (d) Intraoperative.

**Figure 3:**
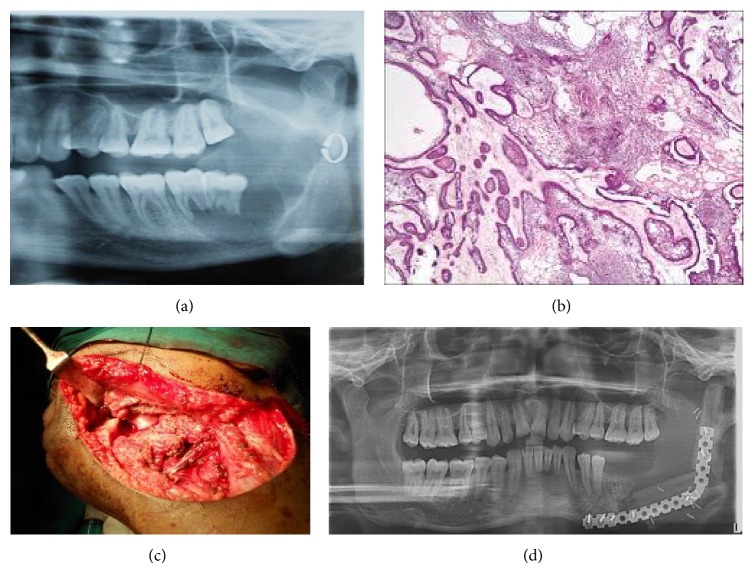
(a) Preoperative OPG. (b) Histopathologic examination. (c) Intraoperative. (d) Postoperative. Fibula with reconstruction plate.

**Figure 4:**
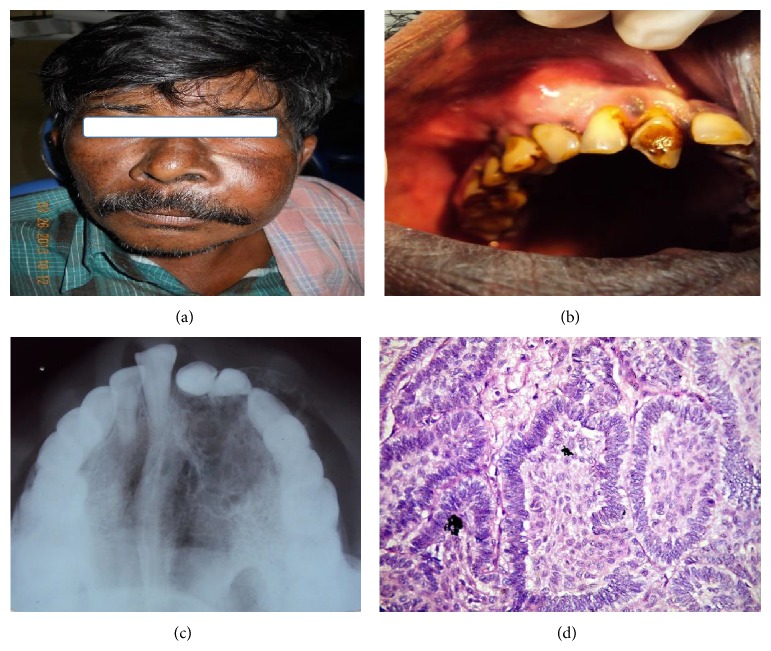
(a-b) Preoperative view. (c) Occlusal radiograph. (d) Follicular type ameloblastoma.

**Figure 5:**
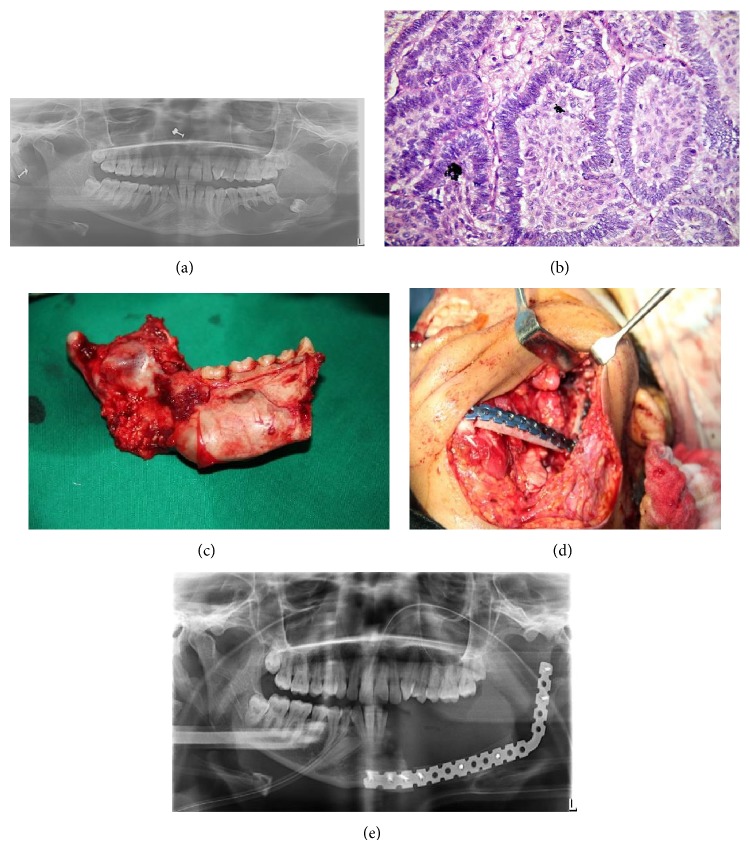
(a) Preoperative OPG. (b) Histopathological examination. (c) Specimen. (d) Rib graft with reconstruction. (e) Postoperative view plate.

**Table 1:** See [6].

Anatomical location	Unicystic lesion	Multicystic/solid lesion
Anterior mandible (cuspid-cuspid)	Curettage/enucleation	Marginal resection Small lesion <3 cm, enucleation with peripheral osteotomy
Posterior mandible (bicuspids-condyle)	Curettage/peripheral ostectomy	Marginal resection without continuity defect (1-2.0 cm margin inferior/posterior border) Segmental resection with continuity defect → thinning of inferior/posterior border
Anterior maxilla (cuspid-cuspid)	Partial maxillectomy	Partial maxillectomy
Posterior maxilla (bicuspid pterygoid plate)	Total maxillectomy	Total maxillectomy

*Note.* The histologic variant types of ameloblastoma should also be considered during treatment planning for all the cases.

**Table 2:** 

Group I: confined to maxilla without involvement of the orbital floor	Partial maxillectomy

Group II: involving orbital floor but not involving periorbital area	Total maxillectomy

Group III: involving orbital contents	Total maxillectomy + orbital exenteration

Group IV: involving skull base	Total maxillectomy + orbital exenteration + skull base resection
